# Combined detection of aneuploid circulating tumor‐derived endothelial cells and circulating tumor cells may improve diagnosis of early stage non‐small‐cell lung cancer

**DOI:** 10.1002/ctm2.128

**Published:** 2020-07-13

**Authors:** Yuanyuan Lei, Nan Sun, Guochao Zhang, Chengming Liu, Zhiliang Lu, Jianbing Huang, Chaoqi Zhang, Ruochuan Zang, Yun Che, Shuangshuang Mao, Lingling Fang, Xinfeng Wang, Sufei Zheng, Jie He

**Affiliations:** ^1^ National Cancer Center National Clinical Research Center for Cancer, Cancer Hospital Chinese Academy of Medical Sciences and Peking Union Medical college Beijing China

**Keywords:** aneuploid circulating tumor‐derived endothelial cells, circulating tumor cells, non‐small cell lung cancer, tumor markers

## Abstract

**Background:**

Many tumor‐derived endothelial cells (TECs) are shed into the blood and turn into circulating TECs (CTECs). Rare circulating non‐hematologic aneuploid cells contain CTCs and CTECs, which are biologically and functionally different from each other. CD31 is one of the most representative endothelial cell (EC) markers, yet CD31 alone is not sufficient to detect malignant CTECs due to the existence of abundant normal ECs in circulation. Aneuploidy of chromosome 8 (CEP8) is an important criterion for the identification of malignant cells. Combined in situ phenotypic and karyotypic characterization, which includes an examination of both protein expression and aneuploid chromosomes, has demonstrated its unique advantage for both effective distinguishing and comprehensive detection of CTCs and CTECs.

**Methods:**

A total of 98 subjects were recruited in the current study, including healthy donors and patients with benign disease and early‐stage non‐small‐cell lung cancer (NSCLC). SE‐iFISH was performed to quantitatively analyze diverse subtypes of aneuploid CD31^+^ CTECs and CD31^−^ CTCs classified upon the ploidy of chromosome 8 and tumor marker expression in the specimens collected from the recruited subjects.

**Results:**

CD31^−^ CTCs primarily consist of triploid CTCs with a small cell size (≤5 µm) and large hyperploid CTCs (≥ pentaploid), whereas CD31^+^ CTECs are mainly comprised of large hyperploid cells. Enumeration of the total numbers of both CTCs and CTECs might help identify malignant nodules with a high sensitivity, whereas quantification of tetraploid CTCs and CTECs specifically exhibited a high specificity for the identification of malignant nodules.

**Conclusions:**

Combined detection of the specific subtypes of aneuploid CD31^+^ CTECs and CD31^−^ CTCs may help to effectively identify malignant nodules with a higher sensitivity and specificity in early stage NSCLC patients.

## Graphical abstract

CTCs primarily consist of triploid CTCs with a small cell size (≤5 µm) and large hyperploid CTCs (≥pentaploid); CTECs are mainly composed of large hyperploid cells; quantification of tetraploid CTCs and CTECs had the potential of identification of malignant nodules.

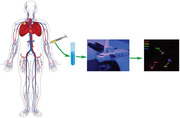

**Abbreviations**
CTC: circulating tumor cells
CTEC: aneuploid circulating tumor‐derived endothelial cell

## 1 INTRODUCTION

There are more than 700 000 new cases of lung cancer in China each year, accounting for 17% of all new cancer cases.^1^ In the past 20 years, the incidence of lung cancer has continued to rise, with a 5‐year survival rate of approximately 16‐18%. Lack of early diagnosis and remote metastasis without warning are important factors in the low 5‐year survival rate of lung cancer. Therefore, it is especially important to find accurate markers for disease discovery and disease progression.

Circulating tumor cells (CTCs) are released into the blood from primary and metastatic foci. They become scattered around the body via blood circulation. When they encounter a suitable microenvironment, they can develop into micrometastases.^2^ They are a very small, phenotypically and functionally diverse group of cells in the blood. CTCs detection is now recognized as the most representative “liquid biopsy” technology, and the integrity and clinical significance of the various types of information it provides far exceed those of other technologies.^3^ In addition to routine diagnosis, staging, efficacy monitoring,^4^ and prognostic effects,^5^, ^6^, ^7^ CTC, with its unique subgroup‐rich advantages, may provide new directions for tumor typing and targeted therapy. However, accurate detection of CTCs has largely limited the clinical application of liquid biopsy strategies.^8^, ^9^

Unlike normal diploid cells, the chromosomes of cancerous cells contain different degrees of variation, often showing a state of aneuploidy. SE‐iFISH technology can simultaneously separate tumor cells from peripheral blood cells by combining the detection of both expression of tumor markers on the surface of tumor cells and chromosomal aneuploidy.^10^ However, we found in a previous study that there were a large number of non‐blood‐derived circulating tumor‐derived endothelial cells (CTECs) in peripheral blood.^11^ These cells had normal, diploid, and aneuploid karyotypes, but the endothelial marker CD31 was strongly positive,^12^ while aneuploid CTECs largely interfered with CTC detection, and their biological characteristics and clinical significance are unclear. During the process of tumor cells detaching from the primary tumor and shedding into the blood, the tumor microenvironment undergoes substantial changes, and the biological characteristics of the tumor cells also change. Aneuploid CTCs and CTECs are two cell populations that are distinguished only by CD31 positivity. Whether this classification is sufficient or whether there is a certain relationship between the two cells needs further verification. Our study confirmed that there were aneuploid CTCs and CTECs in normal individuals, individuals with benign nodules, and patients with different stages of lung adenocarcinoma. We hypothesized that CTCs and CTECs were very different in terms of number, size, surface markers, and chromosomal aneuploidy.

In this article, we further expanded the number of samples, including patients with early‐ and late‐stage lung adenocarcinoma, individuals with benign nodules and normal volunteers, and further explored the biological differences between aneuploid CTCs and CTECs and their number distribution in the development of lung cancer. Analysis of surface tumor markers and changes in chromosome ploidy may provide new ideas for the diagnosis of lung adenocarcinoma. At the same time, subclass analysis of CTCs and CTECs can guide the direction of lung cancer targeted therapy.

## 2 MATERIALS AND METHODS

### 2.1 Patients and samples

All recruited subjects were from the Cancer Hospital of the Chinese Academy of Medical Sciences. Sample processing and preservation were performed by the relevant staff of the thoracic surgery laboratory. Blood sample collection began on November 28, 2017, and ended in December 2019. A total of 98 fresh peripheral blood samples were included, including samples from 18 healthy volunteers, 22 individuals with benign lung nodules, 34 patients with early‐stage adenocarcinoma patients, and 24 patients with advanced lung adenocarcinoma. Normal blood specimens came from healthy volunteers recruited by the thoracic surgery laboratory. The inclusion criteria were age between 45 and 85 years, no precancerous lesions, no tumor history, and no family history of genetic cancer risk. Specimens of benign nodules and early lung adenocarcinoma were obtained from patients undergoing surgery in the Thoracic Surgery Department. Blood samples were collected for enrichment and identification of circulating cells 1 day before surgery. Specimen data based on postoperative pathology were collected. Patients with advanced lung adenocarcinoma were from the Department of Oncology, and the inclusion criteria were untreated (with treatment including radiotherapy and chemotherapy) and newly diagnosed patients.

When collecting peripheral blood from patients, to avoid contamination of epithelial cells, the first 2 mL of blood sample was discarded, or blood samples were taken after routine blood sampling of patients. Blood samples were taken from ACD‐treated blood vessels. After collecting the blood, the tube was immediately inverted eight times to mix the sample. Samples were stored at room temperature protected from light. Samples were processed within 24 h in most cases and not after 48 h.

The study was approved by the Ethics Committee of the Cancer Hospital of the Chinese Academy of Medical Sciences before the study. All patients provided informed consent before blood collection.

### 2.2 Subtraction enrichment of CTCs and CTECs from human peripheral blood

The CTC enrichment kit was purchased from Cytelligen (San Diego, CA, USA), and the procedure was performed according to the manufacturer's protocol. After collecting an appropriate volume of blood, the tube was mixed well and then centrifuged at room temperature for 15 min (200 × *g*). The supernatant up to 5 mm above the brown‐red precipitate was discarded, and CRC buffer was added to dilute the blood cells. Next, 3 mL of the sample density separation liquid was added to a 50 mL centrifuge tube, and the diluted blood cells were added to the upper layer of the sample density separation solution. Next, the mixture was centrifuged at room temperature for 6 min (350 × *g*). After centrifugation, the sample was divided into three layers, and the intermediate transparent or yellow layer and the upper yellow layer were extracted and transferred to a new 50 mL centrifuge tube. Then, 300 µL of washed magnetic beads was added to sample, which was incubated on a shaker at room temperature for 20 min. Finally, the magnetic beads were removed by passing the sample through a magnetic frame, and non‐blood‐derived cells (including tumor cells and endothelial cells) were enriched in the remaining liquid.

### 2.3 Tumor marker immunostaining‐chromosome fluorescence in situ hybridization

The following reagents were purchased from Cytelligen (San Diego, CA, USA). One hundred microliters of the enriched cell solution were added to 2 µL of antigen repair buffer, mixed gently, and shaken, and allowed to stand for 10 min at room temperature. At the same time, the staining solution was prepared. For each slide, 200 µL of blood cell analysis diluent, 1 µL of anti‐CD45 antibody staining solution, 1 µL of anti‐CD31 antibody staining solution, and 1 µL of anti‐vimentin antibody staining solution were used; a sampler was used to gently mix the reagents 10 times to fully distribute the antibodies. The prepared mixture was added to the antigen‐repaired sample and incubated for 20 min at room temperature. The stained cell sample was then washed by CRC washing solution and centrifuged for 5 min (500 × *g*), and the supernatant was discarded, leaving 100 µL. The cells were initially fixed by adding an equal volume of tissue fixative. Then, the samples were dried overnight. The cells on the slide were fixed again the next day, and the fish probe was added for hybridization (hybridization conditions: denaturation at 76°C for 10 min and hybridization at 37°C for 3 h). After the hybridization was completed, the coverslip was slowly removed with the aid of buffer, and washing buffer was added to wash the sample area again to reduce nonspecific staining. The cleaned slide was dried with a hair dryer, 10 µL of DAPI dye solution was added to the center of the specimen, and glass cover slip was applied so that the dye solution fully covered in the entire specimen area. The sample was immediately observed under a fluorescence microscope or preserved at 2‐8°C in the dark.

### 2.4 Image scanning and CTC counting

Image acquiring and analyses of CTCs on the coated and formatted slides (Cytelligen) were performed using an automated Metafer‐iFISH scanning and image analyzing system (Carl Zeiss, MetaSystems, and Cytelligen; ref. 22). Automated *X*‐*Y* scanning with cross *Z*‐sectioning of all cells performed at 1‐mm steps of depth was performed in four fluorescent color channels (DAPI, CD31, CEP8, and CD45). Positive target cells are defined as DAPI^+^, CD45^‐^, and CD31^‐^ with aneuploid Chr8. Automated CTC classification and statistical analyses were performed upon cell size, cell cluster, and chromosome ploidy.

### 2.5 Statistical analyses

Statistical analysis was performed with GraphPad Prism software version 8.0. All data are presented descriptively as the means, medians, or proportions. Two‐tailed Student's *t*‐test was used for statistical comparison between groups; Pearson's correlation analysis was used to determine the correlation between the number of CTCs and CTECs. Statistical significance was defined as *P* < .05.

## 3 RESULTS

### 3.1 Isolation and identification of aneuploid CTCs and CTECs

**FIGURE 1:**
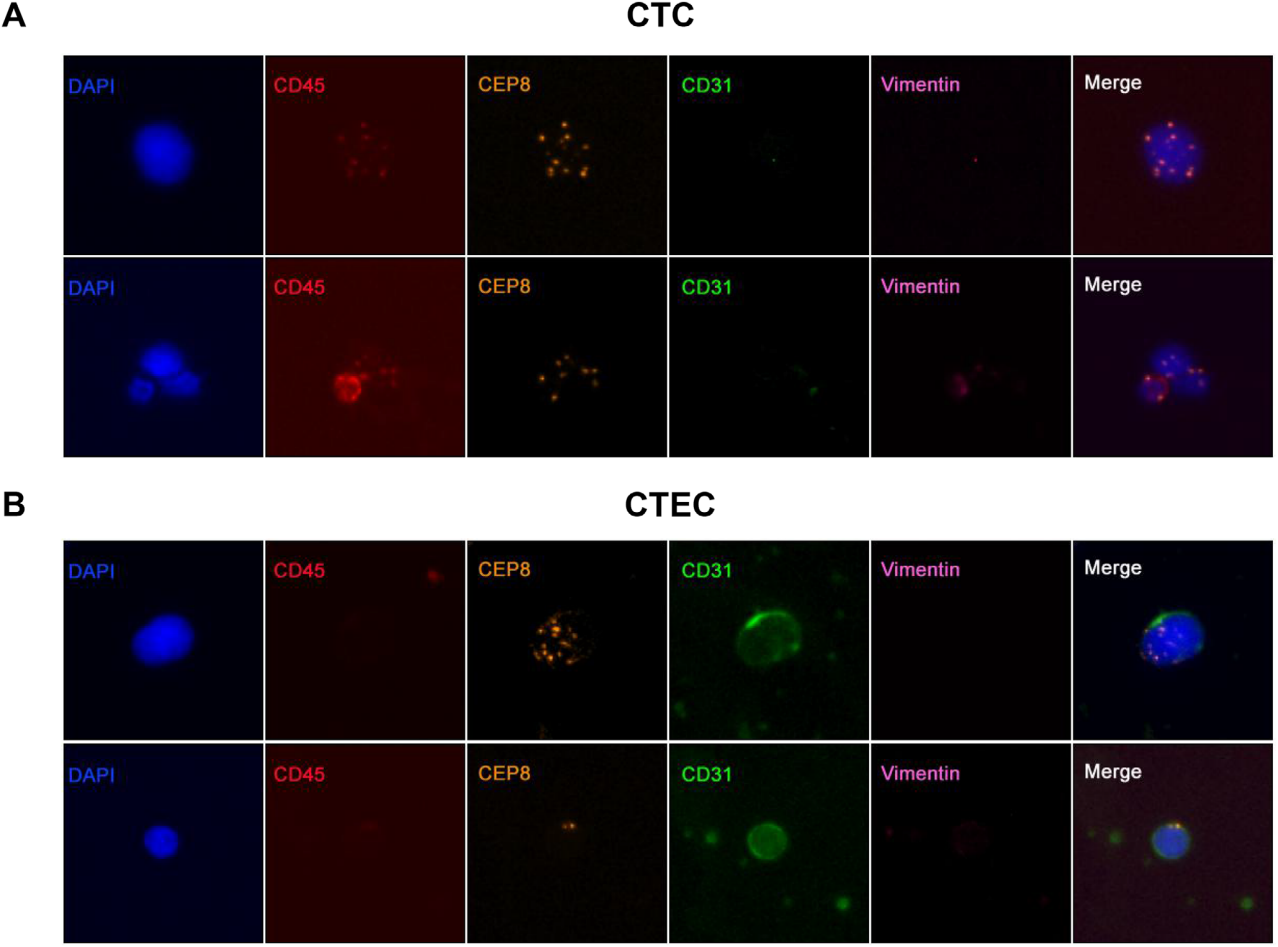
Detection of CTCs and CTECs in the peripheral blood of patients with non‐small‐cell lung cancer by SE‐iFISH (A). Two representative images of CTCs obtained by the SE‐iFISH method. CTC are DAPI^+^/CD45^‐^/CD31^‐^/CEP8^+^, and vimentin represents a tumor marker (and can be either positive or negative). (B). Two representative images of CTECs obtained by the SE‐iFISH method. CTECs are DAPI^+^/CD45^−^/CD31^+^/ aneuploidy^+^

First, we removed blood‐derived cells, such as red blood cells and white blood cells, by centrifugation and phase‐enrichment to achieve high CTC enrichment efficiency, and then we employed the iFISH platform, which combines immunofluorescence staining of tumor proteins (vimentin). With the detection of chromosomal aneuploidy. It can achieve subclassification of CTCs with a high sensitivity and specificity. The CTC identification criteria were as follows (Figure 1A): nuclear DAPI^+^, CD45^‐^, CD31^‐^, chromosome 8 (CEP8) aneuploidy positive, and CTC tumor marker positive or negative. Using this CTC sorting method, we successfully enriched and identified CTCs in the peripheral blood of 98 patients (clinical characteristics are shown in Table 1). When screening aneuploid CTCs under the fluorescence microscope, we also found a large number of aneuploid endothelial cells (CTECs). These cells had the same aneuploidy of CEP8 as tumor cells, but the endothelial cell marker CD31 was strongly positive (Figure 1B).

**TABLE 1:** Clinical characteristics of 98 enrolled individuals with NSCLC

	N (percentage, %)
Age (years, median, range)	60 (43‐84)
Gender	
Male	58 (59.18%)
Female	40 (40.82%)
Disease stage	
Normal	18 (18.36%)
Benign	22 (22.45%)
Early	34 (34.69%)
Late	24 (24.49%)

### 3.2 Distribution of total CTCs and CTECs in patients by stage

**FIGURE 2:**
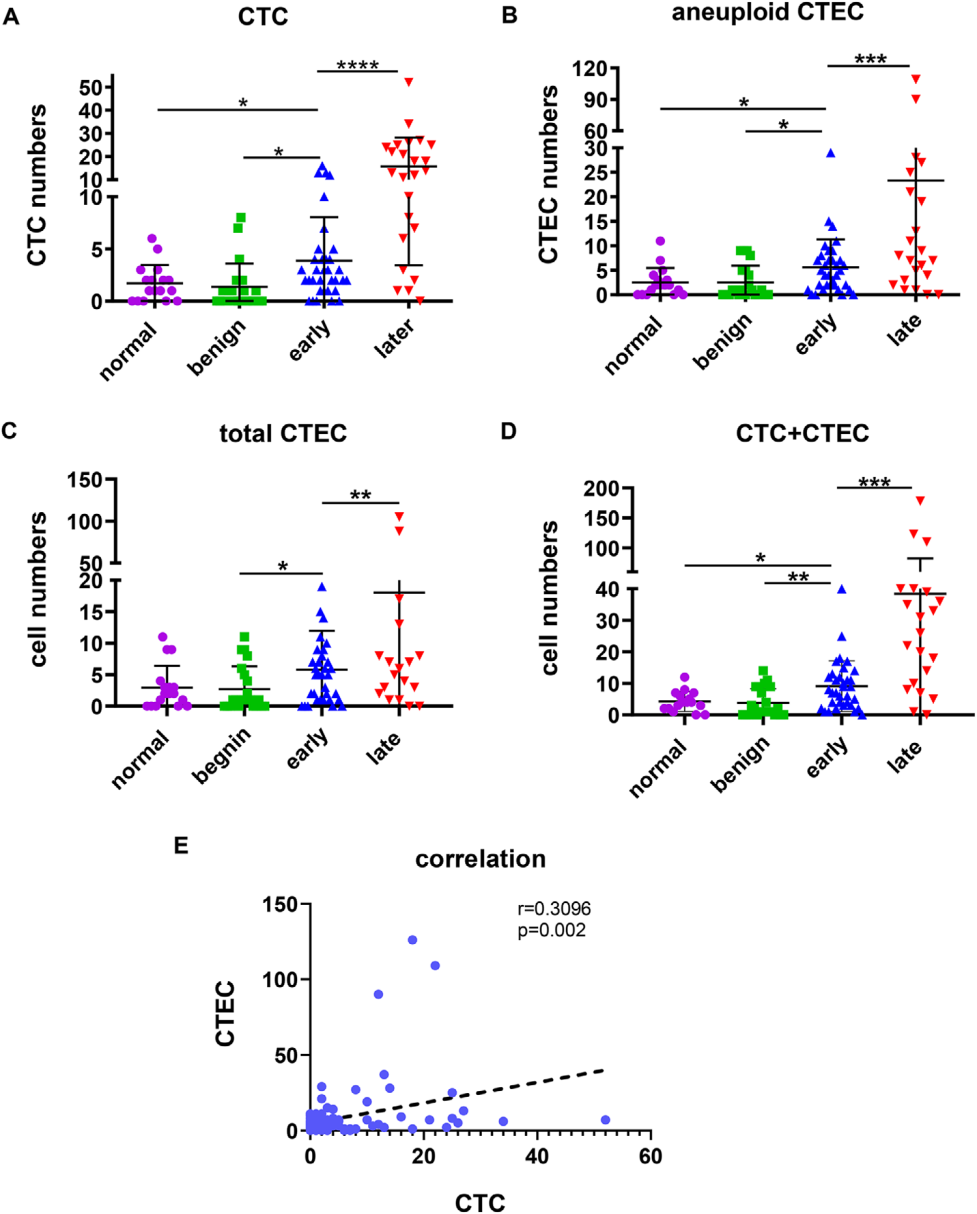
Changes in total CTC and CTEC numbers at different stages of lung cancer development. A, Changes in total CTC counts in normal tissue, benign nodules and early‐ and advanced‐stage lung cancers. B, Changes in total aneuploid CTEC counts in normal tissues, benign nodules and early‐ and advanced‐stage lung cancers. C, Changes in total aneuploid and diploid CTEC counts in normal tissues, benign nodules, and early‐ and advanced‐stage lung cancers. D, Changes in the overall number of CTCs and CTECs in normal tissues, benign nodules, and early‐ and advanced‐stage lung cancers. E, Correlation between dynamics changes in CTC and CTEC numbers. Data are presented as the mean ± SD, ^*^ *P* < .05, ^**^ *P* < .01, ^***^ *P* < .001, ^****^ *P* < .0001. Blank means no significance

We used the iFISH platform to enrich and identify CTCs and CTECs in 17 healthy volunteers, 22 individuals with benign pulmonary nodules, 34 early‐stage lung adenocarcinoma patients, and 22 advanced‐stage lung adenocarcinoma patients. We found that CTCs could be detected in the very early stages of lung cancer. Of the 34 early‐stage lung cancer patients, 29 patients had CTCs, and 17 of these patients had more than three CTCs. Some CTCs could also be identified in normal individual and benign nodule patients. These results indicated that there existed cells in normal people and individuals with benign nodules (aging nontumor cells and cells undergoing apoptosis can also display aneuploidy) that could be mistaken for tumor cells, but the number was mostly less than three, which indicated that the presence of CTCs could still be used to identify benign nodules and early‐stage lung cancer (*P* = .0119; Figure 2A). The number of CTCs in patients with advanced lung cancer was significantly increased compared with that in normal individuals. Likewise, the number of peripheral blood CTCs in patients with advanced lung adenocarcinoma was significantly higher than that in patients with early‐stage lung cancer, individuals with benign nodules and normal controls, and the difference was statistically significant (*P* < .0001; Figure 2A). In addition, we found some CTC‐like circulating tumor‐derived endothelial cells (CTECs) with aneuploidy during the experiment. Their quantitative changes in normal controls, individuals with benign nodules and patients with early‐ and advanced‐stage lung cancer were consistent with those of CTCs (Figure 2B). The number of CTECs in advanced patients was significantly higher than that in normal controls, individuals with benign nodules, and early‐stage lung cancer patients, and CTECs had the same early diagnostic value (*P* = .0287) and tumor‐staging diagnostic value (*P* = .0009) as CTCs. There are normal diploid endothelial cells (CECs) in peripheral blood, and these cells are reported to have diagnostic significance^13^, ^14^, ^15^, ^16^, ^17^ and are closely related to the survival^18^ and relapse^19^ of cancer patients. We counted the number of diploid CECs and found that the number of diploid CECs in peripheral blood was very low; therefore, we observed the dynamic changes in the numbers of both diploid CECs and aneuploid CTECs. The results showed that the number of total CECs was not as good as the number of aneuploid CTECs for identifying early‐ and late‐stage lung adenocarcinoma (Figure 2C). Unexpectedly, we found that there was a correlation between CTCs and aneuploid CTECs in the peripheral blood, and there was a statistically significant difference in their numbers (Figure 2E; *P* = .0003). CTCs and aneuploid CTECs are divided into two categories based on only the expression of CD31 (positive vs negative). As two independent and interrelated markers of tumors, we suspected that the combined consideration of CTCs and CTECs might have more utility than consideration of either population alone. The results showed that the combined consideration of CTCs and aneuploid CTECs had a greater advantage over consideration of either population alone in distinguishing between benign and malignant nodules, and the difference was statistically significant (Figure 2D; *P* = .0069).

### 3.3 Aneuploidy analysis of CTCs and CTECs

**FIGURE 3:**
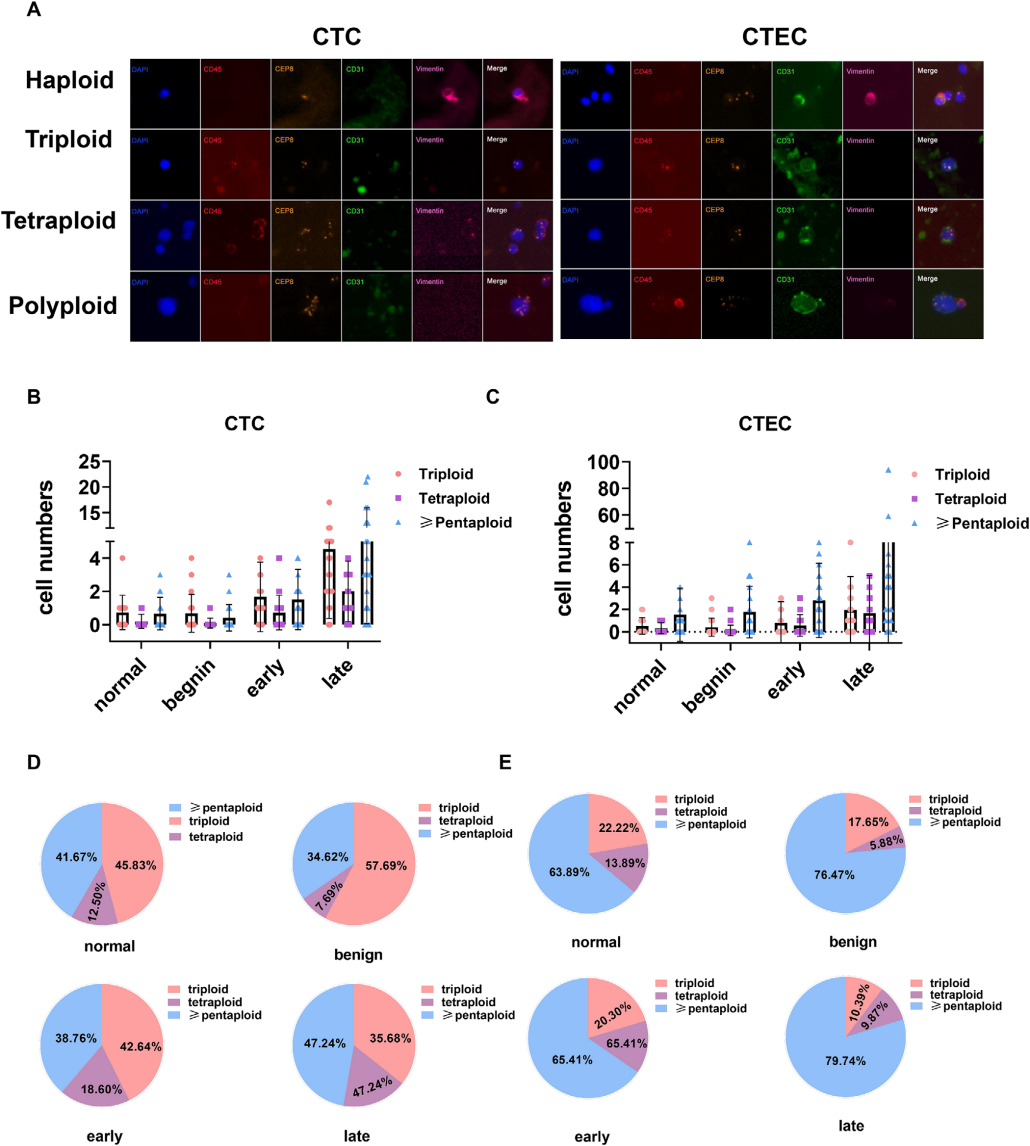
Distribution of haploid and aneuploid CTCs and CTECs in different populations. A, Schematic diagram of chromosomal ploidy in CTCs and CTECs as detected by the SE‐iFISH platform. B and D, Scatter plots and pie charts showing the differences in the distribution of chromosomal ploidy in CTCs in each subgroup (normal tissues, benign nodules, early‐stage lung cancer, and advanced‐stage lung cancer). C and E, Scatter plots and pie charts showing the differences in the distribution of chromosomal ploidy in CTECs in each subgroup (normal tissues, benign nodules, early‐stage lung cancer, and advanced‐stage lung cancer). Data are presented as the mean ± SD, ^*^ *P* < .05, ^**^ *P* < .01, ^***^ *P* < .001, ^****^ *P* < .0001. Blank means no significance

Our study found that both CTCs and CTECs exhibited varying degrees of chromosome aneuploidy, namely, haploid, triploid, tetraploid, and pentaploid karyotypes and beyond. Representative images are shown in Figure 3A. The clinical relevance of different types of aneuploid CTCs has been demonstrated; for example, CTCs with high ploidy have been shown to be associated with tumor resistance and relapse.^20^, ^21^ However, the clinical significance of aneuploid CTECs is still unclear.

We found that chromosome 8 of these sorted CTCs chromosomes 8 showed a pattern. In all normal individuals, individuals with benign nodules, and patients with early‐stage or late‐stage lung cancer, the chromosomal abnormalities predominantly involved polyploidy (the most common aberrations were triploidy and pentaploidy), and only a few had haploidy (Figure 3B,D; due to the very small number of haploids genomes, there was no statistical difference), which is in line with the literature reports that polyploid genetic material is relatively abundant, conducive to the development of malignant biological behavior. The vast majority of CTCs in patients with benign nodules were triploid, but in patients with cancer, especially in patients with advanced stages (Figure 3B,D), pentaploidy was prevalent, suggesting that an increase in genetic material is likely to increase the degree of malignancy. Although CTECs were also dominated by triploidy and pentaploidy, pentaploidy accounted for the vast majority of patients, especially in advanced patients (Figure 3C,E).

### 3.4 Distribution of CTCs and CTECs of different sizes in patients

**FIGURE 4:**
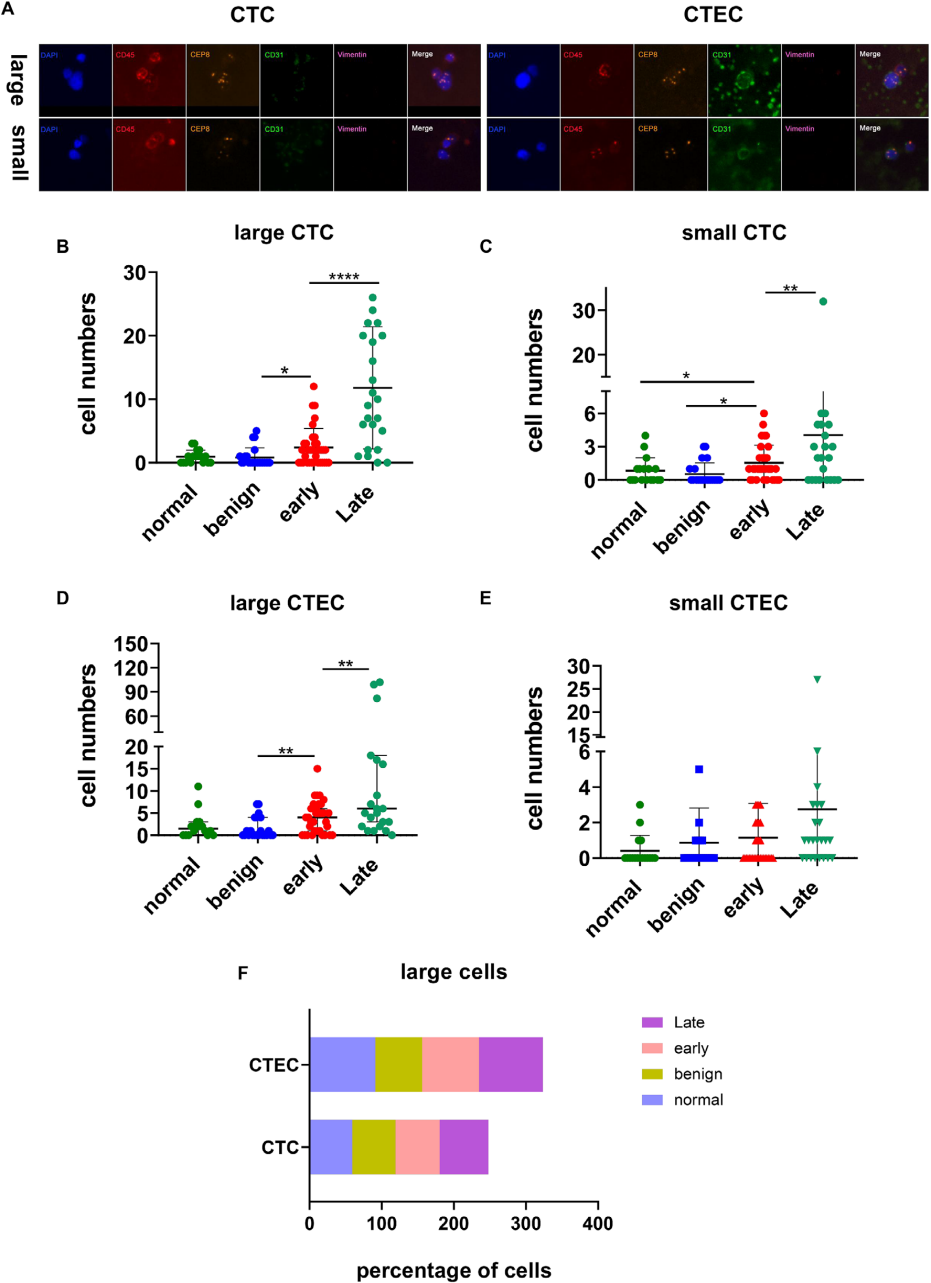
Differences in the numbers and distribution of large and small CTCs and CTECs in different populations. A, Representative pictures of large (≥5 µm) and small (<5 µm) CTCs and CTECs. B, Large CTC quantity distribution among the four categories of samples. C, Small CTC quantity distribution among the four categories of samples. D, Large CTEC quantity distribution among the four categories of samples. E, Small CTEC quantity distribution among the four categories of samples. F, Histogram representing the proportions of large cells (CTCs and CTECs) in different samples. The proportion of large CTECs was relatively high in normal tissues and advanced‐stage cancer tissues, but the number of large CTCs did not vary by stage. Data are presented as the mean ± SD, ^*^ *P* < .05, ^**^ *P* < .01, ^***^ *P* < .001, ^****^ *P* < .0001. Blank means no significance

Peripheral blood CTCs and CTECs vary in size. The definition of large and small cells is generally based on the size of white blood cells (5 µm) as a cutoff, with cells larger than 5 µm defined as large cells, and cells smaller than 5 µm defined as small cells; a representative image is shown in Figure 4A. The biological and clinical significance of CTCs vary between different cancers. For example, small CTCs have been reported to be significantly associated with recurrence and disease‐free survival (DFS) of liver cancer.^21^ However, the correlation between the size of CTECs and their clinical significance is still unclear. Therefore, we separately calculated the distribution of the size of CTCs and CTECs in patients with different stages of cancer. On average, both large and small CTCs and large CTECs (but not small CTECs) had utility in distinguishing between benign and malignant nodules and stages (Figure 4B‐E). However, compared to other cells, large CTCs had an advantage in identifying early‐ and late‐stage lung cancers (Figure 4B; *P* < .0001), while large CTECs were more able to distinguish between benign nodules and early‐stage lung cancers patients (Figure 4D; *P* = .0085). In comparison, small CTECs not only were few in number, but also had poor utility in predicting stage and in diagnosis (Figure 4E). However, when we compared the proportions of large CTCs and large CTECs in different patient populations, we found that there was no difference in the proportion of total circulating cells in the two subcategories, especially large CTCs, in four different populations (Figure 4F).

### 3.5 Chromosome ploidy distribution of CTCs and CTECs of different sizes

**FIGURE 5:**
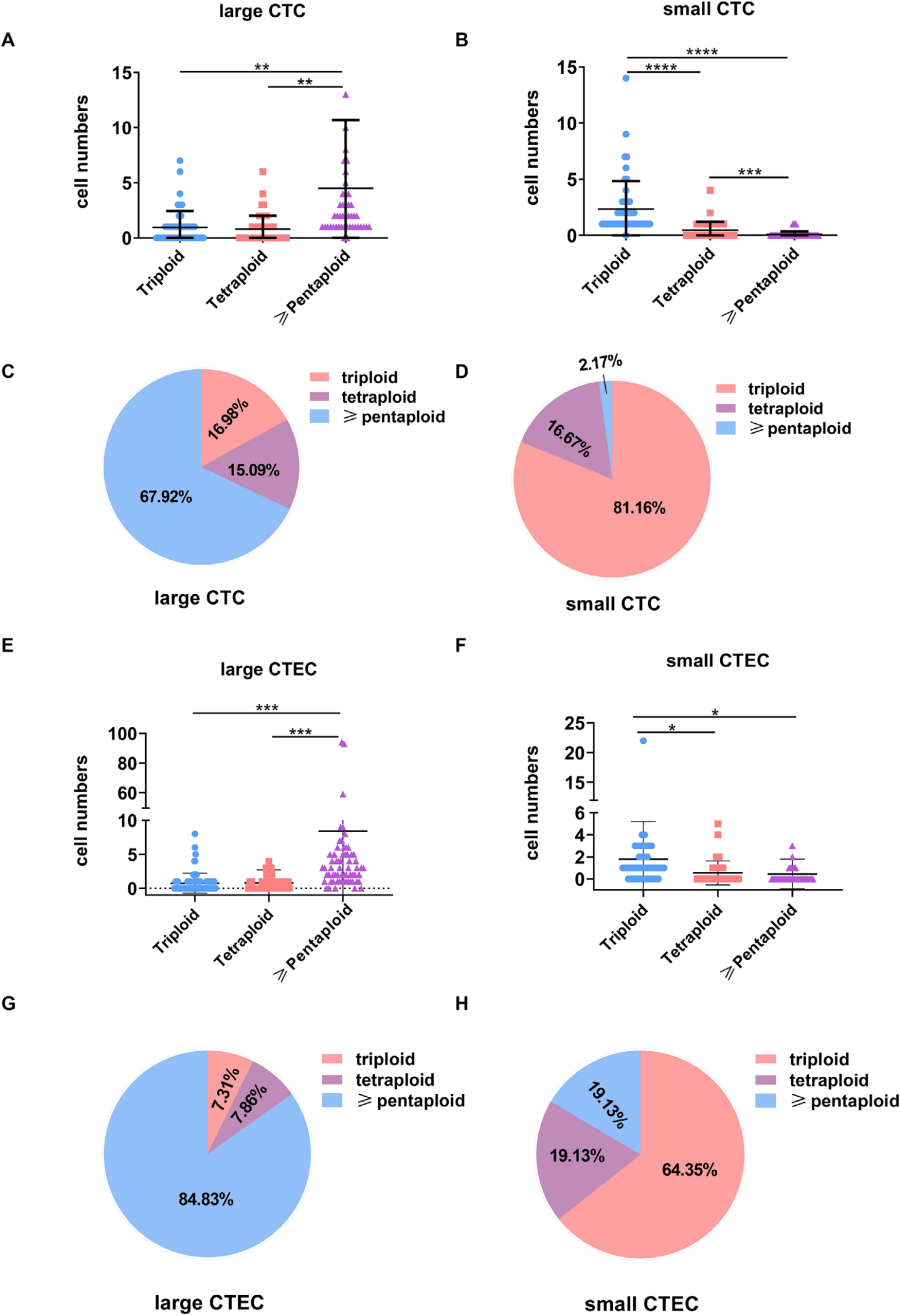
Chromosome ploidy differences between CTCs and CTECs of different sizes. A and E, Chromosome ploidy distribution in large CTCs. B and F, Chromosome ploidy distribution in small CTCs. C and G, Chromosome ploidy distribution of large CTEC. D and H, Chromosome ploidy distribution in small CTECs

Since the chromosomal ploidy of CTC and CTEC subclasses of different sizes has certain clinical significance, we next analyzed the chromosomal ploidy differences of large and small cells. We observed a significant difference in the size of cells with different degrees of ploidy (Figure 5A‐D). According to a cutoff of 5 µm in diameter, CTCs were divided into large cells and small cells, and small cells were mainly triploid, while large cells were mostly pentaploid. The number of cells with tetraploidy was between the numbers of cells with triploidy and pentaploidy, and the distribution of sizes (large or small) was not very different between cells with different degrees of ploidy (Figure 5A,B), but the proportion of tetraploid cells in large CTECs was significantly higher than that in small CTECs (Figure 5C,D). However, as shown in Figure 4, the large cells had significantly more utility in staging and diagnosis than the small cells.

### 3.6 Subclassification of CTCs and CTECs based on both chromosomal ploidy and cell size

**FIGURE 6:**
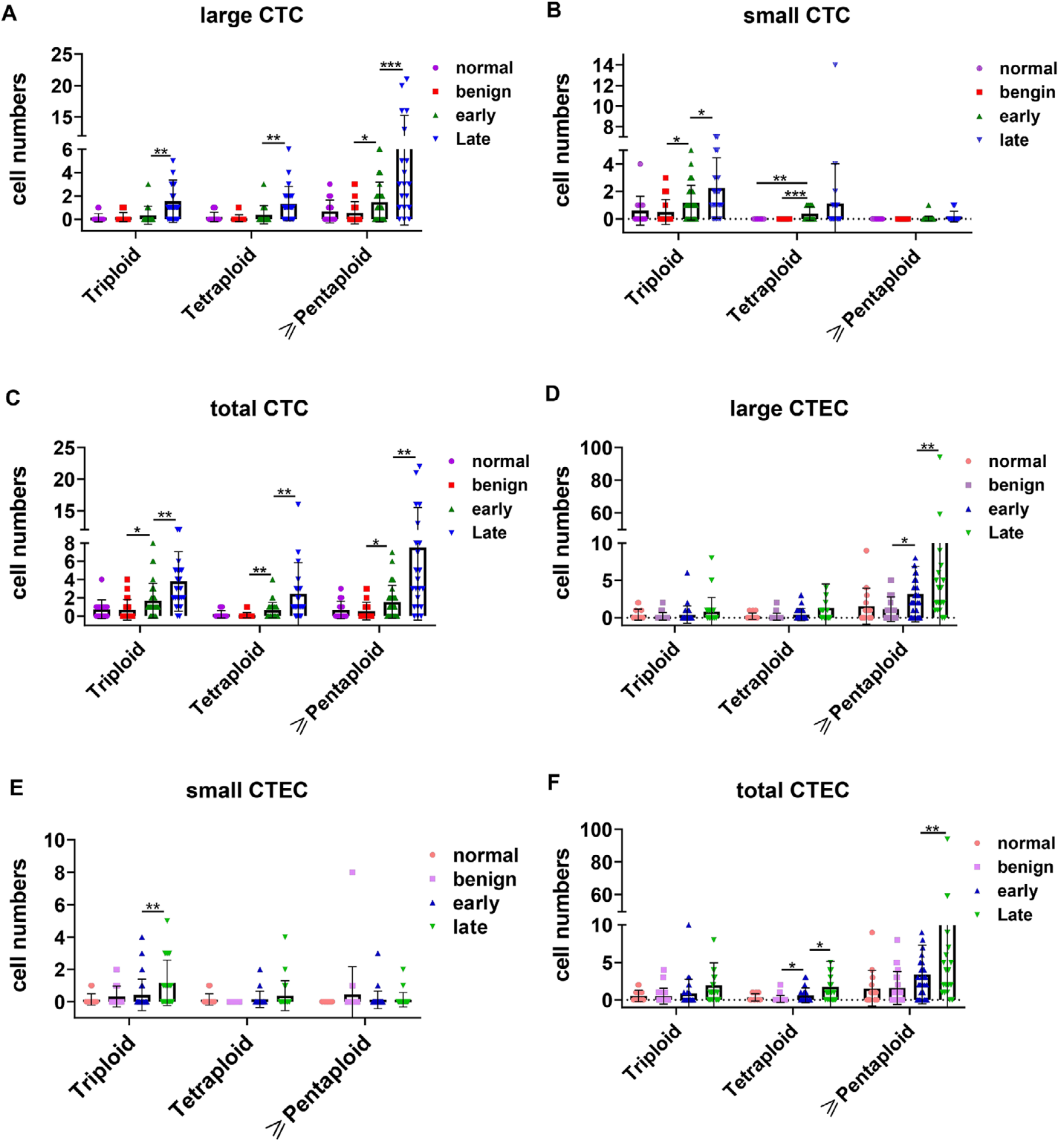
CTC and CTEC size and chromosome ploidy differences across the four different sample types. A, Distribution of chromosomal ploidy of large CTCs in different sample types. B, Distribution of chromosomal ploidy of small CTCs in different sample types. C, Distribution of chromosomal ploidy of total CTCs in different sample types. D, Distribution of chromosomal ploidy of large CTECs in different sample types. E, Distribution of chromosomal ploidy of small CTECs in different sample types. F, Distribution of chromosomal ploidy of total CTCs in different sample types. Data are presented as the mean ± SD, ^*^ *P* < .05, ^**^ *P* < .01, ^***^ *P* < .001, ^****^ *P* < .0001. Blank means no significance

To further understand the significance of the various subtypes of CTCs and CTECs in the early diagnosis and staging of lung cancer, we divided CTCs and CTECs into 24 subgroups based on cell size, chromosome ploidy, and four categories of clinical scenario (normal, benign lesion, early‐stage cancer, and late‐stage cancer) for separate analysis (Figure 6A‐D).

Among the large CTCs, triploid, tetraploid, and pentaploid cells could distinguish between early‐stage and late‐stage cancer patients (*P* < .05), but only the pentaploid cells could distinguish benign nodules and early‐stage cancer (Figure 6A). Compared to cells with other levels of ploidy, pentaploid cells were better able to distinguish between early‐ and late‐stage cancer patients (*P* < .001). For small CTCs, tetraploid cells had obvious early diagnostic significance (*P* < .001), while pentaploid had no advantage in either early diagnosis or staging. However, because tetraploid cells accounted for only a small proportion of the entire CTC population, the results related to their clinical utility may be inaccurate. When we considered the large CTCs and the small CTCs together, the subcategories obtained after the analysis were similar in terms of the distribution of large CTCs in the different stages (Figure 6A,E). Current reports on CTC ploidy tend to combine tetraploidy and pentaploidy into one subgroup, collectively referred to as polyploidy. Compared to triploid cells, hyperploid cells have been identified to have some clinical significance in relation to outcome, such as metastasis, recurrence, and drug resistance.^20^ Therefore, we also tried to combine triploid and tetraploid cells into one subclass, namely, hyperploid cells, and then determined the utility of this new subclass in diagnosis and tumor staging. The hyperploid subclass of large CTCs had no obvious advantage in distinguishing between benign and malignant nodules or distinguishing between early‐stage and late‐stage cancer patients over the individual tetraploidy and pentaploidy subclasses (Figure S1A). Moreover, the ability of small CTCs to identify benign and malignant nodules was lower when they were considered as a single hyperploidy group than when they were considered in separate tetraploidy and pentaploidy subgroups (Figure S1B).

For CTECs, we performed the same analysis. Large pentaploid CTECs could simultaneously distinguish between benign and malignant nodules and patients with early‐ and late‐stage tumors (Figure 6B). Small triploid CTECs could distinguish between early‐ and late‐stage cancer patients but could not distinguish benign nodules from early‐stage cancer patients (Figure 6D). However, after the combination of large and small CTECs into one group, both the tetraploid and pentaploid subgroups had certain clinical significance. The pentaploid group was meaningful for distinguishing only early‐ and late‐stage cancer patients, and the tetraploid group could distinguish between benign nodules and early‐stage lung cancer (Figure 6F). When we combined the tetraploid and pentaploid CTECs into a hyperploid subclass, small hyperploid CTECs could not accurately stage patients (Figure S1D). Large CTECs showed the same utility regardless of whether tetraploid and pentaploid cells were combined into a subgroup; that is, only the pentaploid group had utility, as it could distinguish between benign nodules and early‐stage cancer patient and between early‐stage and late‐stage cancer patients (Figure S1C).

Based on the results from analyzing the CTCs and CTECs, we found that the most advantageous type of CTC for distinguishing between benign nodules and early‐stage cancer patients was small tetraploid CTCs; the most appropriate cells for distinguishing early‐stage and late‐stage patients was the large pentaploid CTCs. For CTECs, the subclass of cells best for distinguishing benign nodules form early‐stage cancer was the combined subclass containing large and small tetraploid cells or large hyperploid cells. However, the best choice for distinguishing between early‐ and late‐stage cancer was large pentaploid CTECs or large hyperploid (tetraploid and pentaploid) CTECs. Finally, in terms of differentiating between benign and malignant nodules as well as between early‐ and late‐stage lung cancer, CTCs were slightly more advantageous than CTECs.

### 3.7 CTECs are not senescent or apoptotic cells, but an active cell population

**FIGURE 7:**
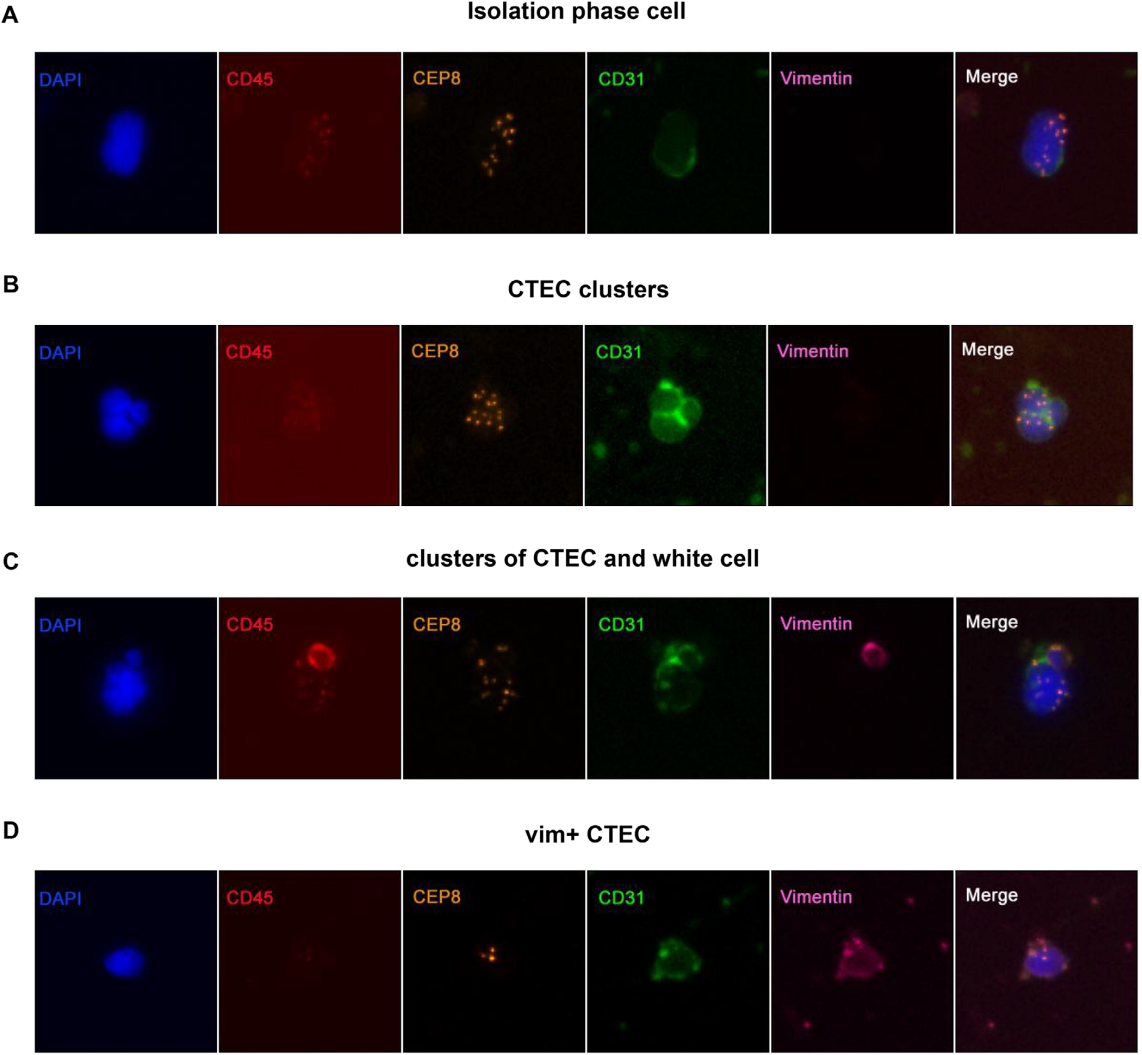
Other biological characteristics of CTECs. A, Aneuploid CTECs in division. B, Cell clumps of aneuploid CTECs. C, Cell clumps formed by aneuploid CTECs and leukocytes. D, Vimentin‐positive aneuploid CTECs

In principle, endothelial cells are nontumor cells, and the genetic material is relatively stable. It only show chromosomal abnormalities under some special stress environment,^22^ while CTEC chromosomes exhibit different degrees of copy number variation,^12^, ^23^ similar to the malignant properties seen in tumor cells. However, despite having many similarities with tumor cells, CTECs are strongly positive for CD31 and are thus classified as a type of endothelial cells. In primary tumors and benign lesions of lung cancer, the expression of CD31 on blood vessels is also uniform (Figure S2A,B). We suspected that these CTECs that entered the blood were old or undergoing apoptosis and therefore showed chromosomal variation. However, we discovered some interesting patterns, such as the phenomenon of cell division in CTECs isolated from peripheral blood (Figure 7A), and CTECs showed characteristics similar to those of tumor cells (Figure 7B) or other types of cells, such as white blood cells (Figure 7C), in line with the literature reports.^24^ The binding of CTCs to leukocytes can promote development of a malignant phenotype by leukocytes,^25^ but the clinical significance of CTECs and leukocyte or tumor cell colocalization is still unclear. In addition, some CTECs also expressed tumor markers such as vimentin (Figure 7D). These phenomena together indicated that aneuploid CTECs might indeed exist as a separate active tumor‐cell‐like population, rather than simply being cells that shed into the blood with age or after death. However, determining whether CTECs have potential malignant behavior and true biological differences from CTCs requires further cellular and molecular experiments, and future studies with single‐cell sequencing will help to understand these issues.

## 4 DISCUSSION

In this article, we explored the biological characteristics of aneuploid CTCs and CTECs and their dynamic changes at different stages of lung cancer development based on chromosome 8 aberration. We found that there were some similarities between the distributions and chromosomal changes of CTCs and aneuploid CTECs. In terms of quantitative changes, both CTCs and aneuploid CTECs could be used as good indicators of lung adenocarcinoma stage. When considering chromosome ploidy, small tetraploid CTCs had advantages in distinguishing early‐stage cancer and benign nodules because small tetraploid CTCs were only found in patients with tumors, while large pentaploid CTCs could better distinguish early‐stage cancer from late‐stage cancer. CTECs were mainly composed of large pentaploid cells. Their main advantage was in distinguishing early‐stage from advanced‐stage lung cancer.

Human blood is rich in biological information, and liquid biopsy can capture cancerous information at an early stage and prevent tumor progression as early as possible. Common liquid biopsy indicators are cfDNAs, RNAs, miRNAs,^26^ and proteins (free proteins or proteins contained in CTCs, exosomes,^27^ or platelets).^28^ cfDNA is commonly used to detect gene mutations, and is suitable for drug resistance and efficacy evaluation, but the blood contains a high abundance of nontumor‐derived cfDNA.^29^ The level of RNA is low in blood, and RNA is not stable. Protein‐based markers generally indicate disease progression with changing levels, but provide limited information reflecting changes in gene levels and tumor heterogeneity. Unlike fragmented nucleic acid and protein markers, aneuploid CTCs and CTECs together form a pair of “cell‐type circulating tumor markers” that are biologically active and rich in a range of tumor markers and complete genetic information. The functions of the two cells are different and complement each other and play an important role in the occurrence and development of tumors.

The utility of detecting tumor cells by chromosomal aneuploidy detection methods (FISH) has been recognized by pathology departments of major hospitals. The use of a chromosome 8 probe (CEP8) for the diagnosis of lung cancer, colorectal cancer, stomach cancer, and liver cancer has been reported in many studies. The iFISH technique, which combines FISH technology with cellular immunofluorescence staining, has been employed for CTC detection in various cancers. For example, Jing Zhang et al found that triploid CTCs could be detected in most newly diagnosed nasopharyngeal carcinoma patients but the number of polyploid (pentaploid or higher ploidy) cells that could be detected in most patients (66.7%) with recurrence and metastasis increased significantly. They also validated that the copy number of chromosome 8 was closely related to chemotherapy efficacy and drug resistance, with tetraploid CTCs being the most related to chemotherapy efficacy, while triploid CTCs were most relevant to drug resistance.^30^ Chromosome 8 aneuploid is also associated with tumor resistance and recurrence in breast cancer, gastric cancer,^31^ and liver cancer. Therefore, an updated subclass analysis of CTCs based on chromosome 8 aneuploidy can allow new breakthroughs in the diagnosis and treatment of tumors.

Another important focus of this article was aneuploid CTECs. In 2004, the Japanese Kyoko Hida team discovered the phenomenon of DNA abnormalities in endothelial cells in tumor tissues in mouse tissue for the first time.^32^ In 2009, they found that in human kidney cancer tissues, approximately 22‐58% of endothelial cells had chromosome 7 and 8 aneuploidy.^33^ Moreover, unlike normal stable diploid cells, these aneuploid endothelial cells had karyotype variation that increased with the number of passages in vitro, suggesting that in the tumor microenvironment, tumor endothelial cells can obtain cytogenetic abnormalities.^33^ The researchers later performed research to speculate that the production of aneuploid endothelial cells was likely caused by hypoxic conditions in the tumor microenvironment.^22^ However, the discovery of aneuploid CTECs in the peripheral blood circulation was only unexpectedly discovered during the detection of CTCs after the rise of iFISH technology (first reported in 2017^24^). In our study, aneuploid CTECs did have obvious enrichment in peripheral blood. At different stages of lung cancer development and progression, their numbers showed dynamic changes, and they were obviously enriched in advanced patients, suggesting that they have clinical significance; similar results were also found in previous studies exploring the distribution of circulating endothelial cells.^34^, ^35^ However, the previous studies were mainly focused on total CECs and did not distinguish between aneuploid and normal diploid cells in detail. We separately analyzed aneuploid cells and diploid cells and found that the number of aneuploid CECs was much higher than that of normal diploid CECs. Whether these aneuploid CECs detached from the aneuploid endothelial cells of the primary tumor is still unclear, and given the nonspecific fluorescence signal in lung cancer tissue, exploring whether there are aneuploid endothelial cells in other parts of lung cancer tissue by traditional immunofluorescence is not feasible, limiting research. Tumor‐derived endothelial cells (TECs) have been proven to promote tumor development in all aspects,^36^, ^37^ but the role of aneuploid CECs in peripheral blood in the development of tumors needs further study.

During the process of tumor cells detaching from the primary tumor and shedding into the blood, the tumor microenvironment undergoes great changes, and the biological characteristics of the tumor cells also change. Aneuploid CTCs and CTECs are two cell populations that are distinguished by CD31 expression, but they are present in similar numbers in different stages of lung cancer development, and their chromosome ploidy distributions are similar. In line with this, an article first reported in 2019 that aneuploid CTECs, like CTCs, can also express a series of tumor markers, such as HER2, PD‐L1, EpCAM, and the stem cell marker CD44.^38^ Simultaneous detection of PD‐L1‐positive aneuploid CTECs and CTCs in addition to other tumor markers brings great convenience to screening patients for immunotherapy.^23^ Moreover, we observed that aneuploid CTECs rarely expressed the interstitial marker vimentin, while this marker was strongly positive on blood vessels. Therefore, we wondered whether this classification method is reasonable or whether there may be some connection between the two kinds of cells. The patterns of marker expression are probably a result of the transformation of cells as they enter the blood, but this hypothesis needs further verification.

This study also has some shortcomings; for example, a small number of CTCs and aneuploid CTECs were also found in normal individuals and individuals with benign nodules. The numbers of these two types of cells need to be further defined to determine the optimum cutoff value for use in the early diagnosis of lung cancer. CTCs and aneuploid CTECs found in normal individuals and individuals with benign nodules may be cells with chromosomal variations caused by inflammation and aging. These CTCs can be classified as “nonauthentic” CTCs, and their degree of biological malignancy may be far less than that of the “true” CTCs; in addition, these cells may be cleared by the immune system at any time, so it is especially important to detect the numbers of such cells at multiple time points. Advances in single‐cell sequencing technology promise to enable the identification of a marker that truly distinguishes between “true” and “nonauthentic” CTCs, making detection more precise.

## 5 CONCLUSIONS

There are significant differences in the number, size, surface markers, and degree of chromosomal aneuploidy between aneuploid CTCs and CTECs in patients with lung adenocarcinoma at different stages. Both types of cells can play a role in the diagnosis and treatment of lung cancer. Future research should focus on identifying the biological characteristics of subclass of aneuploid CTCs and CTECs to guide in‐depth mechanistic research and targeted therapy.

## AVAILABILITY OF DATA AND MATERIALS

The analyzed data sets generated during the study are available from the corresponding author on reasonable request.

## AUTHOR CONTRIBUTIONS

YYL, NS, and JH were responsible for the study concept and design, acquisition, analysis and interpretation of data, and drafting the manuscript. GCZ, CML, LLF, CQZ, and RCZ were responsible for collection of samples. ZLL, JBH, XFW, and SFZ were responsible for processing specimen. YC and SSM were responsible for proofreading.

## ETHICS APPROVAL AND CONSENT TO PARTICIPATE

Informed consent was obtained, in accordance with the Declaration of Helsinki. The study was approved by the Ethics Committee of the Cancer Hospital of the Chinese Academy of Medical Sciences before the study. All patients received informed consent prior to blood collection.

## CONFLICT OF INTEREST

The authors declare that they have no conflict of interest.

## Supporting information

SUPPORTING INFORMATION
