# Supplementary material for: Combined detection of aneuploid circulating tumor‐derived endothelial cells and circulating tumor cells may improve diagnosis of early stage non‐small‐cell lung cancer
Source: Clin Transl Med. 2020 Jul 13;10(3):e128. doi: 10.1002/ctm2.128 (PMC7418803; doi:10.1002/ctm2.128)
Supplement: Supplementary file 1 — SUPPORTING INFORMATION [file CTM2-10-e128-s001.docx]

## Supplemental Data


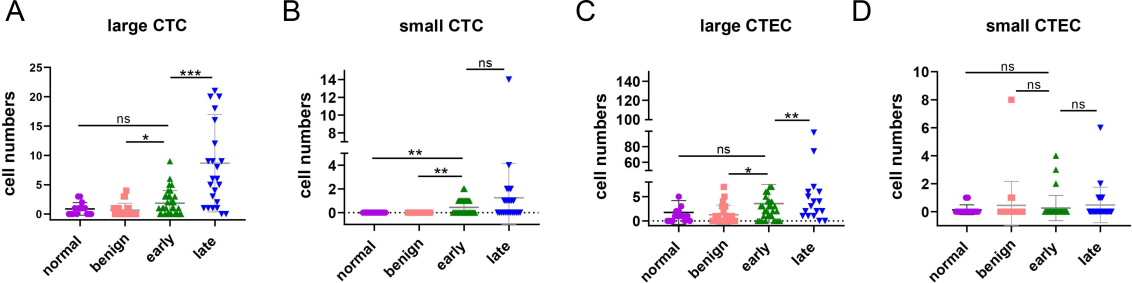


**Figure S1. Subclass analysis of CTCs and CTECs based on chromosomal ploidy and cell size combination. (A).** Changes in large CTCs of hyperploid (tetraploid and pentaploid and above) in different populations; **(B).** Changes in small CTCs of hyperploid (tetraploid and pentaploid and above) in different populations; **(C).** Changes in large CTECs of hyperploid (tetraploid and pentaploid and above) in different populations; **(D).** Changes in small CTECs of hyperploid (tetraploid and pentaploid and above) in different populations; Data are presented as the mean ± SD, *p< 0.05, **p <0.01, ***p < 0.001, ****p < 0.0001. blank means no significance.


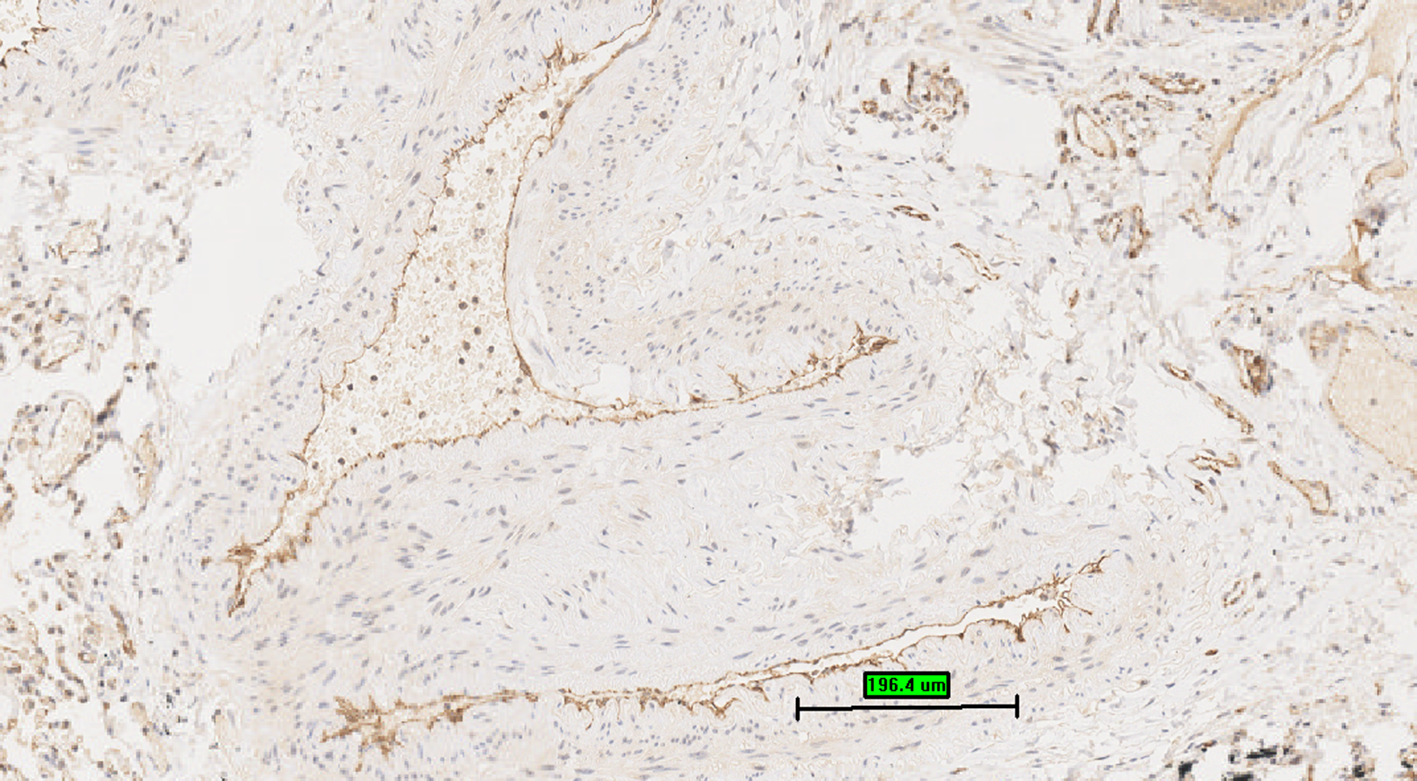

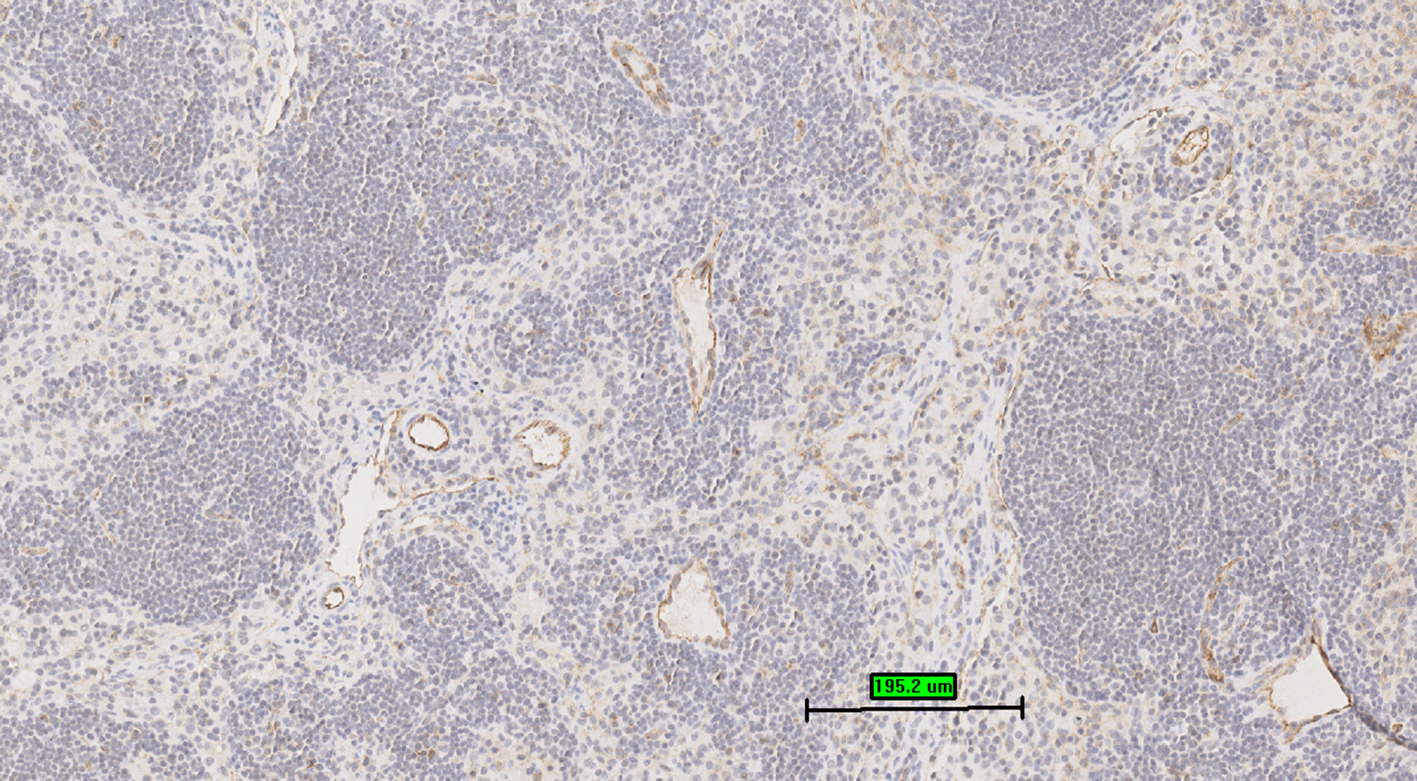


CD31

benign

tumor

**Figure S2. Schematic diagram of immunohistochemistry of CD31 in patients with benign nodules and lung cancer. (A).** Expression of CD31 in benign nodules. **(B).** Expression of CD31 in patients with lung cancer.
